# Time trends of perfluoroalkyl substances in blood in 30-year old Norwegian men and women in the period 1986–2007

**DOI:** 10.1007/s11356-021-13809-6

**Published:** 2021-04-11

**Authors:** Vivian Berg, Torkjel Manning Sandanger, Linda Hanssen, Charlotta Rylander, Therese Haugdahl Nøst

**Affiliations:** 1 Department of Medical Biology, Faculty of Health Sciences, UiT-The Arctic University of Norway, Post Box 6050, NO-9037 Tromsø, Norway; 2 Department of Community Medicine, Faculty of Health Sciences, UiT-The Arctic University of Norway, Tromsø, Norway; 3 NILU, FRAM - High North Research Centre on Climate and the Environment, Tromsø, Norway

**Keywords:** PFAS, Time trends, Biomonitoring, Exposure, Blood concentrations, Reproductive ages, Study design

## Abstract

Biomonitoring studies are helpful tools and can increase our knowledge on time trends in human blood concentrations of PFASs: how they relate to emission trends and the potential prenatal exposure for future generations. In this study, serum was sampled in cross-sections of men and women who were 30 years old in each of the years 1986, 1994, 2001, and 2007 in Northern Norway and analyzed for 23 PFASs. Differences in serum concentrations across sampling years were investigated graphically and with significance testing and compared with those observed in our previous longitudinal study using repeated individual measurements in older men in the same years. The results demonstrate overall increasing blood burdens of PFASs in men and women in reproductively active ages during 1986–2001 and decreases until 2007. However, longer chained PFASs were still increasing in 2007 indicating divergent time trends between the different PFASs, underlining the importance of continued biomonitoring. Comparisons between 30-year-old men and older men within the same population demonstrated variation in time trends in the exact same years, underlining that biomonitoring studies must regard historic exposures and birth cohort effects.

**Supplementary Information:**

The online version contains supplementary material available at 10.1007/s11356-021-13809-6.

## Introduction

Perfluoroalkyl substances (PFASs) have been produced since the 1950s and used in a variety of consumer products due to their chemical properties (Lehmler 2005). The production of PFASs increased from 1966 to the 1990s and remained rather constant from 1990 to 2000 until a phase-out was announced by the 3M Company in 2000 (Paul et al. 2009). This resulted in a rapid drop in production and use of perfluorooctane sulfonate (PFOS) related compounds from the year 2002 (Paul et al. 2009). Several PFASs persist in the environment, have the potential to bioaccumulate, and may have toxic effects in animals and humans. Accordingly, the use of PFOS was restricted in the USA in 2001 (Paul et al. 2009) and in Europe from June 2008 (European Parliament 2006). In the USA, eight of the major perfluorooctanoic acid (PFOA)–producing companies committed to reduce emissions of PFOA and related chemicals by 95% by 2010 through the “PFOA Stewardship Program” (US EPA 2006) and a wide ban of PFOA by EU was implemented the 4^th^ of July 2020 (European Parliament 2020). Additionally, PFOS and PFOA have been classified as persistent organic pollutants (POP) (UNEPA 2015). In Norway, the main emissions of PFOS have been through the use of fire-fighting foams with an estimated use of 57,600 tons in 2005, in addition to 21,500 tons of PFOS and related compounds remaining in stored products (Norwegian Ministry of the Environment 2005). Production, import, and export of PFOA or PFOA-containing products and textiles were banned in Norway from June 1^,^ 2014, as well as the trade in such products produced prior to this date (Norwegian Ministry of the Environment 2013). Following voluntary and regulatory initiatives to minimize PFAS exposures, the global production of PFOS and related chemicals decreased substantially between 1990 and 2000 (Paul et al. 2009). However, the production of longer chain PFASs has likely continued for some years after 2002 (Armitage et al. 2009), and the production of PFOS has continued in China (Zhang et al. 2013). Additionally, the use of the aforementioned PFASs is often substituted with other PFASs, and in 2015 there were 3000 PFASs available on the global market (Kemi 2015).

Despite decreased production and emissions, PFASs reside in environmental compartments and humans are continuously exposed to several of PFASs in their everyday life, primarily through food, but also through water, air, and dermal contact. Concerns for potential health effects of PFAS concentrations in background-exposed populations are therefore still expressed. Biomonitoring data provides a critical tool for evaluating the effectiveness of the voluntary and regulatory initiatives, and most studies report decreasing PFAS concentrations in human serum after the year 2000, with significant declines of PFOS and PFOA (Reviewed in Land et al. 2018). However, trends for the longer chained PFASs like perfluoronanoic acid (PFNA), perfluorodecanoic acid (PFDA), and perfluoroundecanoic acid (PFUnDA) have been inconsistent across populations (Calafat et al. 2007; Glynn et al. 2012; Jain 2018; Kato et al. 2011; Liu et al. 2015; Olsen et al. 2017; Salihovic et al. 2015; Schroter-Kermani et al. 2012). Furthermore, in cross-sectional studies assessing human exposure to PFASs, concentrations have frequently been reported to be positively associated with age and to birth cohorts (Haug et al. 2009). However, in such study designs, age and birth cohort effects are confounded (Glenn 1981; Quinn et al. 2011) and a study on temporal trends from 2002 to 2013 reported higher blood concentrations of certain PFASs in younger compared to older individuals in Australia (Eriksson et al. 2017). Our research group has previously reported intra-individual trends in PFASs concentrations using five repeated measurements during 1979–2007 in serum from 53 aging men (Nøst et al. 2014). In that study, we concluded that a person’s lifetime environmental exposure to PFASs (i.e., the intensity and duration of their environmental exposure) depends on when they were born relative to the time of peak environmental concentrations. To further extend our understanding of the time trends of PFASs in relation to age and birth cohorts, the present study assessed PFAS concentrations in repeated cross-sectional samples of 30-year-olds in the period 1986 to 2007 and compared trends to those in older men in the same geographical region. Furthermore, the age group represented reproductively active ages and could indicate potential prenatal exposure for future generations.

## Material and methods

### Study population

The Tromsø study is a health survey that has occurred every seventh year in the municipality of Tromsø, Northern Norway (summarized by; Jacobsen et al. 2011). We included men and women who were 30 years old when they donated blood in four surveys in the period 1986 to 2007. Participants who had sufficient sample volumes in 1986 (*n* = 29), 1994 (*n* = 29), 2001(*n* = 29), and 2007 (*n* = 30) were included; thus, the present analyses comprised 117 serum samples. Socio-demographic and lifestyle information was extracted from questionnaires. Serum samples were stored at −70 °C until analysis.

### Analytical methodology

A total of 23 PFASs, 13 perfluoroalkyl carboxylic acids (C4-C14, C16, C18), six perfluoroalkyl sulfonates (C4-C8, C10), three fluorotelomer sulfonates (4:2, 6:2, 8:2), and one perfluroalkyl sulfonamide (C8), were analyzed in serum samples at the laboratory at NILU–Norwegian Institute for Air Research, Fram Centre, Tromsø, Norway using sonication-facilitated liquid-liquid extraction with methanol, activated ENVI-carb clean-up (Powley et al. 2005) and analysed by ultrahigh pressure liquid chromatography triple–quadrupole mass spectrometry (UHPLC-MS/MS). The procedures for sample preparation, treatment, and extraction are previously published by Hanssen et al. (2013). The only changes were the volumes used; 2.5 ng internal standard mixture was added to 0.25 ml serum before the addition of 1 ml methanol and 2.0 ng branched PFDA was added as the recovery standard. An aliquot of 100 μl extract was transferred to a vial and mixed with an equal amount of 2 mM aqueous ammoniumacetate (NH4OAc, ≥99%, Sigma-Aldrich, St. Louis, MO, USA) prior to analysis. The analytical method, instrumentation, and reagents are described in detail by Hanssen et al. (2013). Details on compounds, detection rates, and limit of detections (LODs) are presented in Table S1 in the supplemental material.

### Quality control in PFAS analyses

To assure the quality of the PFAS analyses, repetitive analysis of blank samples and reference samples were performed. The laboratory also participates in the Artic Monitoring and Assessment Programme ring test for POPs in human serum, an international comparison program, organized by Institut National de Santé Publique du Québec, Canada (Québec Indspd 2014). The uncertainties of our analyses are within ±15 to 20% of the assigned values, indicated by Interlaboratory comparisons and reference samples. Quantification of the contaminants was performed by the internal standard addition method using isotope-labeled PFASs (Hanssen et al. 2013). In all samples, concentrations of PFAS were within the linear range of the instrument and the calibration curve. A second mass transition served to confirm compound specificity for each compound in the mass spectrometry analyses. The linear PFOS isomers were chromatographically separated from the branched isomers and quantified separately. Summed concentrations of the PFOS isomers (branched PFOS + linear PFOS) were considered in the statistical analyses unless otherwise stated. The LOD was calculated as 3× the signal to noise (background) ratio.

### Data treatment and statistical analyses

All statistical analyses were conducted using STATA (version 16.1; Statacorp, Texas, USA) and a statistical significance threshold of *p* < 0.05 was used. Only PFASs with detection frequencies above 70% were evaluated in the statistical models, and concentrations below the LOD were replaced by LOD/√2. Spearman’s *ρ* values were calculated for correlations and we used the nonparametric test Wilcoxon rank sum test to test differences in PFAS concentrations between sampling years and sex.

## Results

### Study sample characteristics and PFAS concentrations

**Fig. 1:**
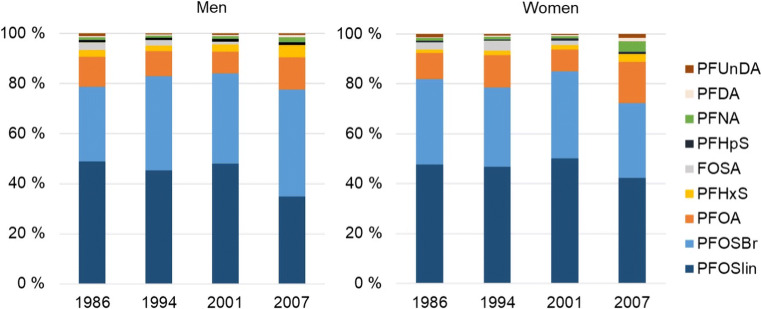
Relative contributions of individual PFASs to their sum (in %) in serum samples in 1986, 1994, 2001, and 2007 in men and women

Characteristics of study participants are presented in Table 1. The proportion of men and women was unequally distributed across the sampling time points and the percentage of women were 55, 62, 48, and 66 % in 1986, 1994, 2001, and 2007, respectively. Median body mass index (BMI) was significantly higher in 30-year-olds in 2001 and 2007 compared to 1986 (Table 1), and BMI was slightly higher in men compared to women at all time points (results not shown). Serum concentrations of PFASs in men and women at each sampling year are presented in Table 2. Eight PFASs were detected in >70% of all the samples. PFOS was detected in highest concentrations in all sampling years followed by PFOA and perfluorooctane sulfonamide (FOSA) (except for in 2007), whereas the magnitude of concentrations of perfluorohexane sulfonate (PFHxS), perfluoroheptane sulfonate (PFHpS), PFNA, PFDA, and PFUnDA varied according to sampling year (Table 2 and Fig. 1).

**Table 1:** Characteristics of 30-year-olds in four population surveys in the Tromsø Study

Sampling year	1986	1994	2001	2007
Age	30	30	30	30
Birth year	1956	1964	1971	1977
Men (*n*)	13	11	15	10
Women (*n*)	16	18	14	20
Median BMI (5, 95 percentiles)	23 (20, 27)	24 (20, 36)	25 (19, 32)	25 (20, 33)
No. of child births (% of all women)^a^				
0	4 (25)	9 (50)	6 (43)	12 (60)
1	3 (19)	3 (17)	4 (29)	4 (20)
2	9 (56)	5 (28)	3 (21)	4 (20)
3	-	1(5)	-	-
4	-	-	1 (7)	-
Birth year of children	NA^b^	1985–1994	1991–2000	1995–2007
Median birth year first child	NA^b^	1988	1994	2000
Median total breastfeeding months	NA^b^	9	8	11

^a^For 1986 and 1994, there were 1 and 3 women, respectively, that did not report any explicit number of children nor any details of any child birth in the questionnaires. These were assumed not to have children. ^b^*NA*, not available; (*n*), number of subjects. *BMI*, body mass index (kg/m^2^)

**Table 2:** Concentrations (ng/ml) of perfluoroalkyl substances in serum from 30-year-olds in four different surveys of the Tromsø study

Women	1986 *n* = 16			1994 *n* = 18			2001 *n* = 14			2007 *n* = 20		
	Median	Min	Max	Median	Min	Max	Median	Min	Max	Median	Min	Max
FOSA	0.54	0.15	4.61	1.15	0.30	3.70	0.61	0.17	1.65	-	-	-
PFHxS	0.26	0.07	0.91	0.52	0.15	1.23	0.63	0.25	7.74	0.47	0.05	0.97
PFHpS	0.10	<LOD	0.36	0.15	<LOD	0.58	0.26	<LOD	1.39	0.13	<LOD	0.47
ΣPFOS	15.3	9.15	34.9	22.7	6.52	53.2	28.1	8.51	65.7	10.6	3.88	24.4
PFOS linear	8.82	5.37	18.0	13.2	4.20	30.2	16.6	4.99	35.8	6.06	2.53	14.2
PFOS branched	6.34	3.42	16.9	8.96	2.32	23.0	11.5	2.97	29.9	4.31	1.34	10.2
PFOA	1.97	0.94	3.98	3.64	1.08	6.97	2.89	0.68	5.31	2.37	0.81	7.22
PFNA	0.21	<LOD	0.39	0.30	<LOD	0.40	0.29	<LOD	0.75	0.59	0.23	1.47
PFDA	0.05	<LOD	0.13	0.14	<LOD	0.26	0.17	0.06	0.43	0.21	0.06	0.59
PFUnDA	0.26	<LOD	0.81	0.18	<LOD	0.40	0.16	<LOD	0.70	0.23	<LOD	1.12
ΣPFAS	19.3	11.2	43.1	29.2	8.92	64.9	34.8	10.5	76.0	16.0	6.58	31.8
Men	1986 *n* = 13			1994 *n* = 11			2001 *n* = 15			2007 *n* = 10		
	Median	Min	Max	Median	Min	Max	Median	Min	Max	Median	Min	Max
FOSA	0.84	0.17	3.44	1.09	0.15	4.49	0.59	0.24	2.78	-	-	-
PFHxS	0.74	0.39	3.40	1.19	0.70	2.22	1.42	0.61	3.85	1.29	0.67	3.25
PFHpS	0.24	<LOD	0.33	0.44	0.22	0.75	0.49	0.25	0.89	0.30	<LOD	1.46
ΣPFOS	21.7	10.1	47.4	41.1	24.8	63.9	41.6	23.9	53.9	20.1	14.5	49.1
PFOS linear	13.3	5.31	26.0	22.2	13.8	37.1	23.2	12.5	36.3	9.28	7.75	30.5
PFOS branched	8.13	4.77	21.3	18.5	9.87	27.0	17.5	10.5	27.1	11.4	6.77	19.5
PFOA	3.31	1.02	7.84	4.92	3.36	15.1	4.16	2.69	7.72	3.44	1.77	7.72
PFNA	0.33	0.22	0.64	0.46	0.23	0.63	0.54	0.36	0.76	0.53	0.41	1.09
PFDA	0.08	<LOD	0.14	0.19	0.11	0.49	0.26	0.12	0.49	0.23	0.07	0.51
PFUnDA	0.33	0.16	1.02	0.22	0.10	0.63	0.31	<LOD	0.78	0.22	<LOD	0.68
ΣPFAS	25.8	12.8	61.1	52.5	34.1	74.0	49.4	30.2	63.5	26.4	19.9	59.3

*FOSA*, perfluorooctane sulfonamide; *PFHxS*, perfluorohexane sulfonate; *PFHpS*, perfluoroheptane sulfonate; *∑PFOS*, sum perfluorooctane sulfonate; *PFOA*, perfluorooctanoic acid; *PFNA*, perfluoronanoic acid; *PFDA*, perfluorodecanoic acid; *PFUnDA*, perfluoroundecanoic acid; *ΣPFASs*, sum perfluoroalkyl substances

### Differences in PFAS concentrations between sexes

**Fig. 2:**
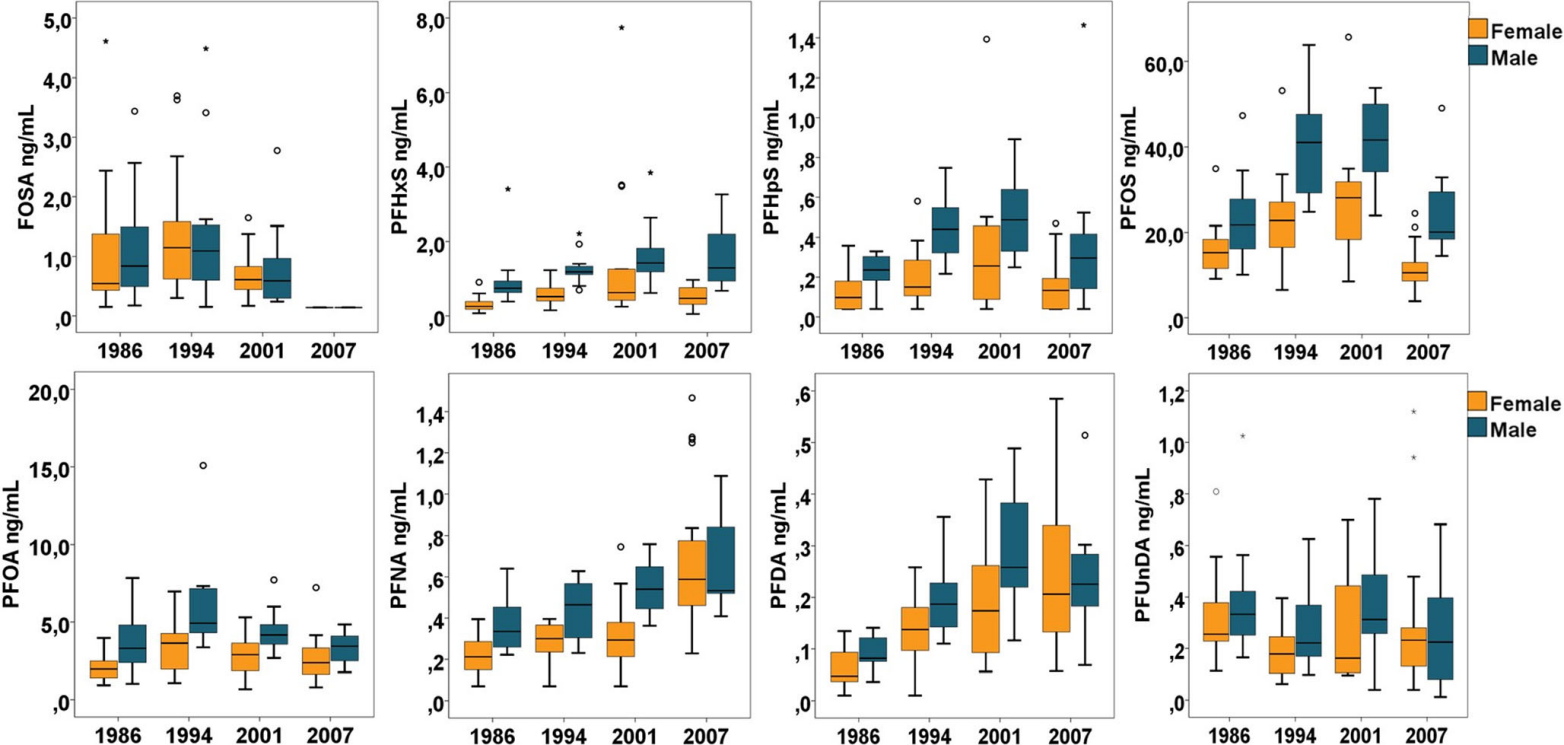
PFAS concentrations in serum from 30-year-old men (blue boxes) and women (orange boxes) in the years 1986, 1994, 2001, and 2007. Ranges on the *Y*-axis vary across components. Boxes represent the 25th–75th percentiles, horizontal lines represent the median, whiskers indicate 1.5 times the length of the interquartile range above and below the 75th and 25th percentiles, respectively, and outliers are represented as data points

Concentrations in women and men in the different sampling years are depicted in Fig. 2. Women had lower concentrations of PFASs compared to men at all time points (Fig. 2 and Table 2), and the differences were significant (Wilcoxon rank sum test *p*-values <0.05) for PFHxS (in 1986, 1994, and 2007), PFHpS (in 1986 and 1994), PFOS (in 1994, 2001 and 2007), and PFOA (in 1994) and PFNA (in 2001). The % branched PFOS were lower for women compared to the men at all time points (not significantly in 1986) and were 39, 43, 40, and 42 % for women and 45, 45, 44, and 52 % for men in 1986, 1994, 2001, and 2007, respectively. In 1986 and 2004, multiparous women had lower median concentrations of FOSA compared to women who had not given birth (not statistically tested), whereas there were no differences in the other PFAS concentrations according to parity in these years. Multiparous women had lower median concentrations of FOSA, PFHxS, PFHpS, PFOS, and PFOA in 2001 and lower concentrations of all detected PFASs in 2007 compared to women who had not given birth (Table S2).

### PFAS concentrations across surveys

The median summed (Σ) PFAS concentrations increased from 1986 to 2001 in women, and from 1986 to 1994 in men (Table 3). Concentrations in 2007 relative to 2001 decreased by half for both sexes (54% and 47% for women and men, respectively). For the individual compounds, median concentrations of PFHpS, PFHxS, and PFOS increased from 1986 to 2001 in both men and women, followed by a decline from 2001 to 2007. PFOA followed the same pattern but demonstrated peak concentrations in 1994. In comparison, concentrations of PFNA and PFDA were higher in 2007 compared to 2001 in women and were rather unchanged across the same years in men. For PFUnDA, no specific time trends between sampling years were observed. FOSA had the third highest concentrations in 1986 and 1994 but concentrations dropped significantly from 1994 to 2001 (*p* < 0.05), and FOSA was not detected above LOD in any of the samples in 2007.

**Table 3:** Difference in median concentrations (ng/ml) of perfluoroalkyl substances in 30-year-olds between survey years

Compound	Gender	1986–1994			1994–2001			2001–2007		
		Difference in ng/ml	Annual change ng/ml	% change	Difference in ng/ml	Annual change ng/ml	% change	Change in ng/ml	Annual change ng/ml	% change
FOSA	Women	0.61	0.087	113	−0.54	−0.077	−47	-	-	-
PFHxS	Women	0.26	0.037	100	0.11	0.016	21	−0.16	−0.023	−25
PFHpS	Women	0.05	0.007	50	0.11	0.016	73	−0.13	−0.019	−50
ΣPFOS	Women	7.40	1.057	48	5.40	0.771	24	−17.5	−2.500	−62
PFOS linear	Women	4.38	0.626	50	3.40	0.486	26	−10.5	−1.506	−63
PFOS branched	Women	2.62	0.374	41	2.54	0.363	28	−7.19	−1.027	−63
PFOA	Women	1.67	0.239	85	−0.75	−0.107	−21	−0.52	−0.074	−18
PFNA	Women	0.09	0.013	43	−0.01	−0.001	−3	0.30	0.043	103
PFDA	Women	0.09	0.013	180	0.03	0.004	21	0.04	0.006	24
PFUnDA	Women	−-0.08	−0.011	−31	−0.02	−0.003	−11	0.07	0.010	44
ΣPFASs	Women	9.9	1.414	51	5.60	0.800	19	−18.8	−2.686	−54
FOSA	Men	0.25	0.036	30	-0.50	−0.071	−46	-	-	-
PFHxS	Men	0.45	0.064	61	0.23	0.033	19	−0.13	−0.019	−9
PFHpS	Men	0.20	0.029	83	0.05	0.007	11	−0.19	−0.027	−39
ΣPFOS	Men	19.4	2.771	89	0.50	0.071	1	−21.5	−3.071	−52
PFOS linear	Men	8.90	1.271	67	1.00	0.143	5	−13.9	−1.989	−60
PFOS branched	Men	10.4	1.481	128	-1.00	−0.143	−5	−6.10	−0.871	−35
PFOA	Men	1.61	0.230	49	-0.76	−0.109	−15	−0.72	−0.103	−17
PFNA	Men	0.13	0.019	39	0.08	0.011	17	−0.01	−0.001	−2
PFDA	Men	0.11	0.016	138	0.07	0.010	37	−0.03	−0.004	−12
PFUnDA	Men	−0.11	−0.016	−33	0.09	0.013	41	−0.09	−0.013	−29
ΣPFASs	Men	26.7	3.814	103	−3.10	−0.443	−6	−23.0	−3.286	−47

*FOSA*, perfluorooctane sulfonamide; *PFHxS*, perfluorohexane sulfonate; *PFHpS*, perfluoroheptane sulfonate; *∑PFOS*, sum perfluorooctane sulfonate; *PFOA*, perfluorooctanoic acid; *PFNA*, perfluoronanoic acid; *PFDA*, perfluorodecanoic acid; *PFUnDA*, perfluoroundecanoic acid; *ΣPFASs*, sum perfluoroalkyl substances

The correlations of PFASs within sampling years are presented in Table S3. Correlations between PFOS and PFOA decreased across the period (*r*_*s*_ = 0.70, 0.76, 0.63, and 0.51 in 1986, 1994, 2001, and 2007, respectively), whereas correlations between PFNA, PFDA, and PFUnDA increased during the period (*r*_*s*_ = 0.60, 0.71, 0.73, and 0.75 between PFNA and PFDA in 1986, 1994, 2001, and 2007, respectively).

### Comparing time trends in different study designs

**Fig. 3:**
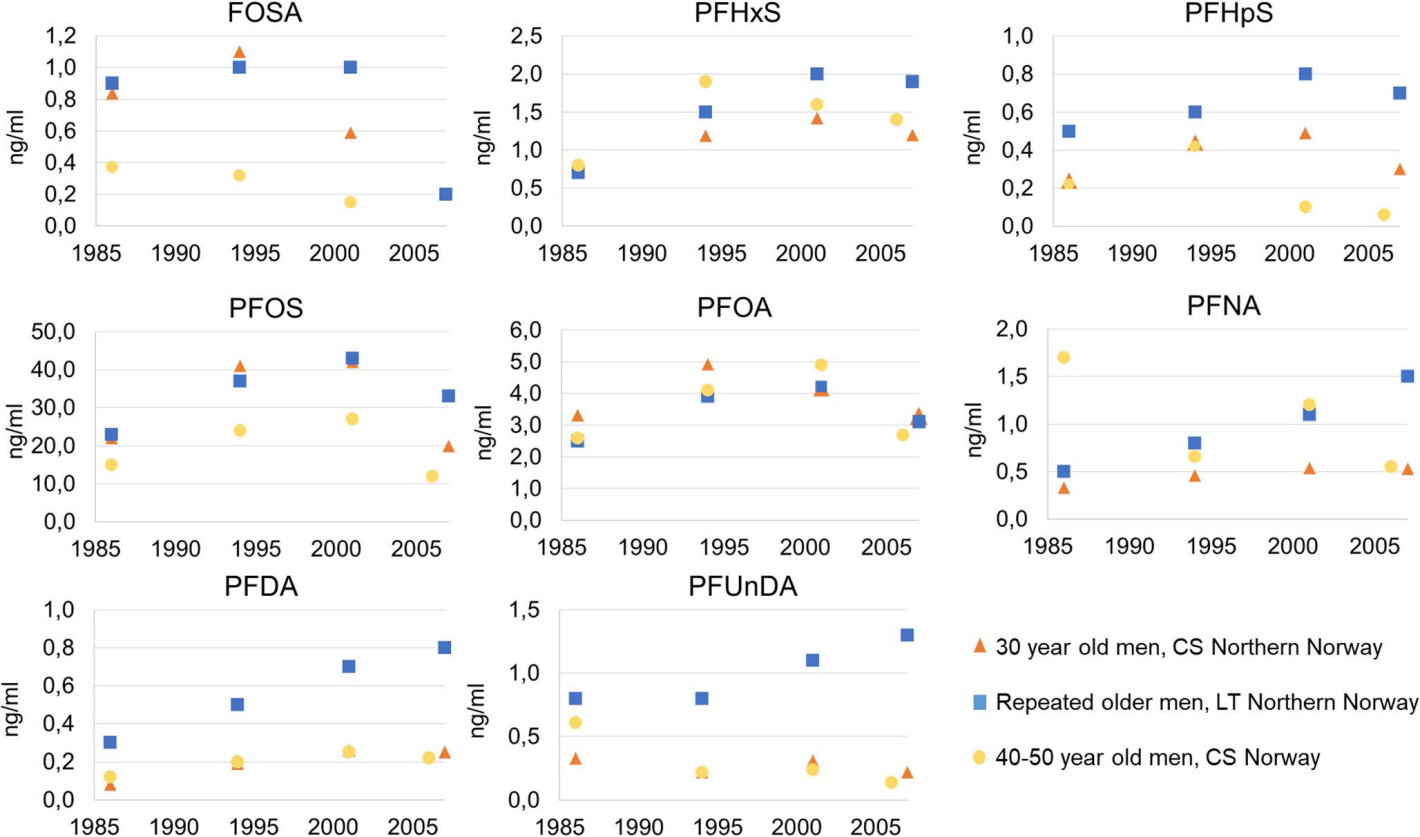
Comparing PFAS concentrations (ng/ml) across sampling year (*y*-axis) in the two Norwegian repeated cross-sectional (CS) sampling of 30-year-old men (present study) or 40–50-year-old men (Haug et al. 2009), and the longitudinal (LT) study with repeated individual measurements in older men (median age increase from 50 to 71 between 1986 and 2007) (Nøst et al. 2014)

The overall time trends in the period 1986 to 2007 were similar between 30-year-old men and the older men from the same population surveys (Fig. 3), whereas the median concentrations of several PFASs and the relative increase and decrease between surveys differed. The observed relative decreases from 2001 to 2007 in 30-year-old men were −9%, −38%, and −52% for PFHxS, PHFpS, and PFOS, respectively and the corresponding decreases for the same sampling years in the older men were −6%, −13%, and −22%. PFNA, PFDA, and PFUnDA concentrations decreased in the 30-year-olds but increased in the older men, whereas both study sample sets decreased similarly in PFOA concentrations. Concentrations and time trends in men in the present study were comparable to results from a previous study from Norway, including repeated cross-sectional samples of pooled blood from men in the age group 40–50 years old (Haug et al. 2009); however, PFOS concentrations in the 30-year-olds in Northern Norway were higher than concentrations in the 40–50-year-old men at all time points (17 versus 15; 27 versus 24; 33 versus 27; and 13 versus 12 ng/ml PFOS in 1986, 1994, 2001, and 2007 respectively). Figure 3 displays only the comparison between men as no comparable study was found that included only women.

## Discussion

This study reports individual concentrations of PFAS in men and women in reproductively active ages during a period of 22 years before and after peak emissions. We demonstrate that concentrations of several PFASs in 30-year-old men and women in Northern Norway have decreased considerably since the year 2000. The cross-sectional serum time trends of PFASs in men and women were increasing from 1986 to 2001 followed by a decrease to 2007 for PFHxS, PFHpS, and PFOS. Furthermore, PFOA concentrations were the highest in 1994 and decreased towards 2007. However, increasing concentrations were observed for PFNA, PFDA, and PFUnDA, and this result stresses the importance of continued biomonitoring of human exposure to these environmental pollutants. Concentrations of PFASs were lower in women as compared to men at all time points, and in 2001 and 2007, concentrations were lower in multiparous women compared to women who had not given birth. The age group of 30-year-olds in this study was chosen to represent reproductively active ages in Northern Norway. This study group was targeted to provide relevant monitoring results for human exposure but especially to indicate prenatal exposure for future generations. Accordingly, our study demonstrates that early life exposures to PFASs through breastfeeding have been extensively reduced since the year 2000. In the present study, similar to previous studies, our findings suggest that human blood concentrations reflect historic production and use of PFASs in the subsequent years (Paul et al. 2009).

The present results demonstrate a mean annual decrease in PFOS from 2001 to 2007 of 8.9% in women and 7.4% in men, whereas PFOA decreased annually by 2.5% in women and 2.1% in men from 1994 to 2007. These results are in line with results from both longitudinal and cross-sectional studies from the same period (Glynn et al. 2012; Haug et al. 2009; Nøst et al. 2014; Schroter-Kermani et al. 2012). Glynn and colleagues investigated temporal trends of PFASs in pooled serum from Swedish primiparous women in the period 1996 to 2010. They reported a yearly decrease in PFOS and PFOA of 8.4% and 3.1%, respectively. In the present study, PFNA and PFDA concentrations increased annually from 1986 to 2007 by 8.6 % and 15% in women, and 2.9% and 8.9% in men, indicating a continued exposure to these PFASs. However, recent studies show divergent temporal trends for the longer chained PFASs. A study from Denmark investigated temporal trends in nulliparous women from 2008 to 2013 and demonstrated decreasing concentrations of the longer chained PFASs throughout the period (Bjerregaard-Olesen et al. 2016), whereas a study from Australia demonstrated increasing concentrations from 2002 to 2013 for some of the longer chained PFASs (Eriksson et al. 2017).

The overall time trends in the period 1986 to 2007 were similar between 30-year-old men and in older men from the same population sampled in the same years (Nøst et al. 2014) in that PFOS, PFHxS, PFHpS, PFOA, and FOSA increased until peak emissions around 2001 and decreased thereafter, and PFNA, PFDA, and PFUnDA increased throughout the study period. Concentrations of PFHxS, PFHpS, and especially PFNA, PFDA, and PFUnDA were higher in the older men as compared to the younger men, whereas concentrations of PFOS, PFOA, and FOSA were similar across these two age groups. Median concentrations of PFOS were similar in 30-year-old men and the older men in 1986, 1994, and 2001, whereas lower for the younger men in 2007 (20 versus 33 ng/ml, respectively). Concentrations of PFOA were comparable in 1986 (3.3 versus 2.5, respectively) and in 1994 (4.9 versus 3.9 ng/ml, respectively) and similar in 2001. Accordingly, the 30-year-old men in 1986, 1994, and 2001 were born before or in the period of increasing production of PFASs, and should theoretically have experienced the same average PFAS exposure as the older men (born between 1925 and 1950). The decrease in concentrations of PFOS between 2001 and 2007 was larger in the 30-year-old men compared to the older men. Those who were 30 years old in 2007 were born in 1977, and have, based on marked increases in estimated emissions of PFASs in these years (Paul et al. 2009), experienced different exposure scenarios from birth compared to the older study group born in 1925–1950. In accordance with such interpretation of biomonitoring trends in relation to past emission and use, comparing intra- and inter-individual polychlorinated bisphenol (PCB) and organochlorine pesticide (OCP) concentrations in the same study samples (Nøst et al. 2019), we demonstrated that PCB and OCP concentrations in 30-year-olds decreased with decreasing exposures and were lower in each sampling year compared to the older men (Nøst et al. 2013) due to higher historic exposure in the older birth cohorts. Furthermore, the decreasing concentrations between 2001 and 2007 in 30-year old men were more pronounced as compared to the trends observed in the aging men which was also observed for PCBs and OCPs (Nøst et al. 2019). For example, the median decrease in PFOS between 2001 and 2007 in 30-year-old men was 52% compared to 22% in the aging men. However, the design differs between these observations as in the study with repeated serum samples in the same individuals; the birth cohorts were fixed and the age increased chronologically, whereas in the present study, the age is fixed and the birth cohorts are more recent between each population survey. Therefore, the differences in decreases between the age groups is likely a result also of the different study designs as one can expect smaller differences in one individual over time (intra-individual difference) compared to between different individuals (inter-individual difference). Also, a steeper decline in several PFASs in 30-year-old men compared to older men may partly be explained by a decrease in intake of fish in the younger men, as trends has shown a decreasing intake of fish in younger people in Norway over the last years (Norwegian Directorate of Health 2019). Still, PFOS in our study sample was higher at all time points compared to concentrations in pooled serum from 40- to 50-year-old men from all over Norway (Haug et al. 2009). Overall, our results demonstrate that changes in human exposure to PFASs across calendar years reflect change in emissions and environmental concentrations, thus individual concentrations of PFASs depend on birth cohort and exposure history.

Median concentrations in women were significantly lower than for men for several PFASs in all four surveys. This has been observed in most studies comparing concentrations of PFASs in men and women (Coakley et al. 2018; Goralczyk et al. 2015) and is probably largely due to the elimination routes through childbirth, breastfeeding, and menstruation in women (Harada et al. 2005; Haug et al. 2011; Rush et al. 2018; Wong et al. 2014). Similar, in men, the percentage of branched PFOS was higher compared to women at all time points, and this has been reported in other studies as well (Liu et al. 2015; Rylander et al. 2009; Zhang et al. 2014). This indicates different accumulation and elimination patterns of PFOS in men and women and may be due to a higher elimination rate of branched PFOS by placental transfer during pregnancy (Beesoon et al. 2011; Hanssen et al. 2010). As toxicokinetics for several PFAS have been demonstrated to be similar across sexes (Harada et al. 2005), observed differences in concentrations between sexes are likely due to elimination routes of PFASs present only in women. This is supported by that parity, breastfeeding duration, and the use of contraceptives have been important predictors of PFAS concentrations in women (Berg et al. 2014; Brantsæter et al. 2013; Haug et al. 2011; Rush et al. 2018).

Overall, the observed PFAS concentrations in our study sample are in line with the concentration ranges of PFASs in the period 1986–2007 reported for both women and men by other studies from overlapping sampling periods (Berg et al. 2014; Eriksson et al. 2017; Haug et al. 2009; Jain 2018; Kato et al. 2011; Nøst et al. 2014; Schroter-Kermani et al. 2012). However, concentrations in the 30-year-olds in the present study were slightly higher compared to concentrations reported from other studies including this age group in comparable sampling years in Norway. For example, pregnant women aged 30–34 years from the Norwegian Mother, Father and Child Cohort Study (MoBa) sampled in 2003–2004 (Brantsæter et al. 2013) had lower median concentrations of PFOS and PFOA compared to the 30-year-old women in Tromsø in 2001 (11.9 versus 28.1 and 1.91 versus 2.89 ng/ml, respectively) and may be due to higher exposure related to higher intake of fish in people living in Northern Norway. Accordingly, the Norwegian national dietary survey (Norkost 3) reported a fish intake in the age group 30–39 years of 106 and 91 g per day in men and women from Northern Norway, respectively, versus 71 and 46 g per day for men and women in the total population (Totland et al. 2012). Regarding potential prenatal exposure for future generations and potential adverse effect, EFSA has reported a lowest benchmark dose (BMLD10) of 17.5 ng/ml for the sum of PFNA, PFOA, PFHxS, and PFOS in serum in 1-year-old children. This corresponds to a long-term maternal exposure of 0.63 ng/kg bodyweight per day and breastfeeding the child for 12 months. This would correspond to a pre-pregnancy maternal serum concentration of 6.9 ng/ml at the age of 35 for these PFASs. In comparison, the median concentrations of these four PFAS in the 30-year-old women in the present study were 18, 28, 35, and 15 ng/ml in 1986, 1994, 2001, and 2007, respectively. Thus, the observed concentrations indicated that children were exposed to concentrations above the recommended level for safe intake of these PFASs in the period 1986–2007 and that PFASs exposure for children is still a concern although concentrations of PFASs are decreasing.

In the present study, multiparous women had lower median concentrations of all PFASs in the years 2001 and 2007 compared to nulliparous women and these results are in accordance with the MoBa study who reported that parity was the strongest predictor for concentrations of PFASs in their study in pregnant women sampled in 2003–2004 (Brantsaeter et al. 2013). Except for FOSA, there were no differences in PFAS concentrations according to parity in 1986 and 1994. There were few nulliparous women among the women included in the first two study years, which may have affected these differences. These differences may also be influenced by the relative importance of childbirth and breastfeeding as elimination sources for PFASs differ in pre- and post-ban periods. As the time trends of PFOS and PFOA in human blood demonstrate sharp declines between 2001 and 2003 (Harada et al. 2005; Harada et al. 2004; Inoue et al. 2004; Olsen et al. 2003; Olsen et al. 2005), childbirth and breastfeeding after this period would result in a more pronounced effect on maternal blood concentrations compared to before the peak. Accordingly, in a cohort of northern Norwegian pregnant women, nulliparous women had higher PFAS concentrations compared to multiparous women in 2007–2009 (for example, 10 ng/mL versus 4.5 ng/mL in median PFOS, respectively) (Berg et al. 2014), whereas in a study from Sweden, Ode et al. (2013) did not observe differences in concentrations between parity groups in the years 1978 to 2001.

PFOS, PFOA, and PFHxS were detected in all samples across all sampling years, whereas the detection frequencies of FOSA, PFHpS, PFNA, PFDA, and PFUnDA varied between the years. PFOS contributed most to the summed PFASs in all years, and decreased the most from 2001 to 2007, followed by PFHpS, PFHxS, PFOA, and PFDA. This result demonstrates the dominance of PFOS, as also observed in other studies (Berg et al. 2014; Brantsaeter et al. 2013; Calafat et al. 2006; Calafat et al. 2007; Glynn et al. 2012; Haug et al. 2009). Concurrently, correlations within the shorter chained PFASs (C_6_–C_8_) decreased during the sampling period, while correlations between the longer chained (C_9_–C_11_) increased. The compositional patterns and correlations likely reflect the time passed since peak emissions, as exposure sources have been diluted along with time and according to half-lives of the compounds (Nøst et al. 2017). Hence, increasing correlations between the longer chained PFASs may indicate continued productions and common exposure sources as well as longer half-lives, whereas decreased correlations between the sulfonates may indicate eliminated or reduced exposures and subsequent diluted exposure routes. Finally, as FOSA and other preFOS can degrade to PFOS, this might explain why the percentage of branched PFOS were increasing and at its highest in 2007 in both men and women. Accordingly, Glynn et al. (2012) reported a significant increasing trend of branched PFOS between 2000 and 2010.

Fifteen of the targeted PFASs were not detected in the study sample although several of these have been reported in blood from other populations from the same study period (Gebbink et al. 2015; Glynn et al. 2012; Miaz et al. 2020). For example, detectable concentrations of PFBA, PFHxA, PFHpA, PFTriDA, PFBS, 6:2 FTS, and 8:2 FTS have been reported in primiparous women from Uppsala, Sweden (Miaz et al. 2020). However, sample volumes extracted and detection rates differed between studies and could explain why it is not detected in this study. Still, the low concentrations indicated demonstrates the dominance of the PFASs presented in this study and that the exposure to other PFASs is low in Northern Norway.

This study included a limited number of samples and included both men and women where the women were both nulliparous and parous. This limited the statistical power and led to a higher assumed variance, and we chose not to perform statistical testing of differences in PFAS concentrations according to parity. Furthermore, individual intake of dietary variables was not available across the study period and we could not assess if the time trends were affected by changes in dietary habits across the study period. Also, information of previous breastfeeding periods was not available for all women in the first sampling year. An important strength of this study is that we have individual and not pooled data, enabling us to assess individual variation in PFAS concentrations within populations with the same age and over periods of time.

## Conclusions

Median serum concentrations of PFASs in 30-year-old men and women from four different sampling years reflected overall historic production and use of PFASs in this period. The results demonstrated a decrease in blood burdens of PFASs in men and women of reproductively active ages in the past two decades. This study and our previous study in aging men demonstrate differences in PFAS concentrations and time trends according to age group, birth cohorts, and study designs, and the importance of voluntary and regulatory initiatives for these substances.

## Supplementary Information

ESM 1 (DOCX 60 kb)

## Data Availability

The data that support the findings of this study are available from the Tromsø Study registry, but restrictions apply to the availability of these data, which were used under license for the current study, and so are not publicly available. However, data are available from the authors upon reasonable request and with the permission of the Tromsø Study.
