# Supplementary material for: Time trends of perfluoroalkyl substances in blood in 30-year old Norwegian men and women in the period 1986–2007
Source: Environ Sci Pollut Res Int. 2021 Apr 11;28(32):43897–907. doi: 10.1007/s11356-021-13809-6 (PMC8357677; doi:10.1007/s11356-021-13809-6)
Supplement: Supplementary file 1 — (DOCX 60 kb) [file 11356_2021_13809_MOESM1_ESM.docx]

**Supplemental Material**

**Time trends of perfluoroalkyl substances in 30 year old men and women in the period 1986-2007**

Vivian Berg^1^, Torkjel Manning Sandanger^2,3^, Linda Hanssen^2^ Charlotta Rylander^3^ and Therese Haugdahl Nøst^2,3^

^1^Department of medical biology, Faculty of health sciences, UIT The arctic university of Norway, Tromsø, Norway

^2^NILU, FRAM - High North Research Centre on Climate and the Environment, Tromsø, Norway

^3^Department of community medicine, Faculty of health sciences, UIT The arctic university of Norway, Tromsø, Norway

**Table S1.** **Overview of analyzed compounds, limit of detections (LODs) and detection frequencies**

| **Compound** | **Acronym** | **LOD ng/mL** | **%>LOD 1989** | **%>LOD 1994** | **%>LOD 2001** | **%>LOD 2007** |
| --- | --- | --- | --- | --- | --- | --- |
| Perfluorobutane sulfonate | PFBS | 0.05 | 0 | 0 | 0 | 0 |
| Perfluoropentane sulfonate | PFPeS | 0.05 | 0 | 0 | 0 | 0 |
| Perfluorohexane sulfonate | PFHxS | 0.04 | 100 | 100 | 100 | 100 |
| Perfluoroheptane sulfonate | PFHpS | 0.06 | 72 | 93 | 93 | 77 |
| Perfluorooctane sulfonate | PFOS | 0.10 | 100 | 100 | 100 | 100 |
| Perfluorodecane sulfonate | PFDS | 0.10 | 0 | 3 | 13 | 3 |
| Perfluorobutanoate | PFBA | NA | NA | NA | NA | NA |
| Perfluoropentanoate | PFPeA | NA | NA | NA | NA | NA |
| Perfluorohexanoate | PFHxA | 0.08 | 14 | 0 | 0 | 0 |
| Perfluoroheptanoate | PFHpA | 0.07 | 7 | 0 | 0 | 0 |
| Perfluorooctanoate | PFOA | 0.05 | 100 | 100 | 100 | 100 |
| Perfluorononanoate | PFNA | 0.10 | 93 | 93 | 97 | 100 |
| Perfluorodecanoate | PFDA | 0.02 | 93 | 93 | 100 | 100 |
| Perfluoroundecanoate | PFUnDA | 0.06 | 93 | 100 | 93 | 90 |
| Perfluorododecanoate | PFDoDA | 0.29 | 10 | 7 | 3 | 10 |
| Perfluorotridecanoate | PFTrDA | 0.10 | 0 | 0 | 0 | 0 |
| Perfluorotetradecanoate | PFTeDA | 0.05 | 0 | 0 | 0 | 0 |
| Perfluorohexadecanoate | PFHxDA | 0.10 | 0 | 0 | 0 | 0 |
| Perfluorooctadecanoate | PFODA | 0.10 | 0 | 0 | 0 | 0 |
| Perfluorooctane sulfonamide | FOSA | 0.20 | 100 | 100 | 100 | 0 |
| 4:2 fluorotelomer sulfonate | 4:2 FTS | 0.10 | 0 | 0 | 0 | 0 |
| 6:2 fluorotelomer sulfonate | 6:2 FTS | 0.10 | 0 | 0 | 0 | 0 |
| 8:2 fluorotelomer sulfonate | 8:2 FTS | 0.10 | 0 | 0 | 0 | 0 |

NA= Not available. There were only 1 MS-transition available and quantified concentrations could not be confirmed.

**Table S2. Concentrations (ng/ml) of PFASs across surveys according to sex and parity.**

|  |  | **1986^a^** | | | **1994^b^** | | | **2001^c^** | | | **2007^d^** | | |
| --- | --- | --- | --- | --- | --- | --- | --- | --- | --- | --- | --- | --- | --- |
|  | **LOD** | **Median** | **Range** | **%>LOD** | **Median** | **Range** | **%>LOD** | **Median** | **Range** | **%>LOD** | **Median** | **Range** | **%>LOD** |
| **FOSA** | **0.2** | **0.57** | **0.15-4.61** | **100** | **1.15** | **0.15-4.49** | **100** | **0.59** | **0.17-2.78** | **100** | **-** | **-** | **0** |
| ♂ |  | 0.84 | 0.17-3.44 | - | 1.10 | 0.15-4.49 | - | 0.59 | 0.27-2.78 | - | - | - | - |
| ♀ |  | 0.54 | 0.15-4.61 | - | 1.15 | 0.30-3.70 | - | 0.61 | 0.17-1.65 | - | - | - | - |
| 0-para |  | 1.14 | 0.15-2.44 | - | 1.21 | 0.62-3.63 | - | 0.61 | 0.43-0.99 | - | - | - | - |
| 1-para |  | 0.27 | 0.18-0.54 | - | 0.63 | 0.46-2.68 | - | 0.61 | 0.44-1.38 | - | - | - | - |
| 2-para |  | 0.57 | 0.47-4.61 | - | 0.47 | 0.30-3.70 | - | 0.19 | 0.17-0.83 | - | - | - | - |
| **PFHxS** | **0.04** | **0.44** | **0.07-3.40** | **100** | **0.75** | **0.15-2.22** | **100** | **1.20** | **0.25-7.74** | **100** | **0.73** | **0.05-3.26** | **100** |
| ♂ |  | 0.74 | 0.39-3.40 | - | 1.19 | 0.70-2.22 | - | 1.42 | 0.61-3.85 | - | 1.2 | 0.67-3.26 | - |
| ♀ |  | 0.26 | 0.07-0.91 | - | 0.52 | 0.15-1.23 | - | 0.63 | 0.25-7.74 | - | 0.46 | 0.05-0.97 | - |
| 0-para |  | 0.29 | 0.07-0.91 | - | 0.59 | 0.15-1.23 | - | 1.11 | 0.25-7.74 | - | 0.73 | 0.30-0.97 | - |
| 1-para |  | 0.23 | 0.21-0.40 | - | 0.58 | 0.43-1.00 | - | 0.54 | 0.42-0.87 | - | 0.33 | 0.05-0.46 | - |
| 2-para |  | 0.28 | 0.15-0.61 | - | 0.42 | 0.33-0.59 | - | 0.53 | 0.38-3.51 |  | 0.23 | 0.15-0.48 | - |
| **PFHpS** | **0.06** | **0.18** | **LOD-0.36** | **72** | **0.27** | **LOD-0.75** | **93** | **0.33** | **LOD-1.39** | **93** | **0.16** | **LOD-1.46** | **77** |
| ♂ |  | 0.24 | LOD-0.33 | 92 | 0.44 | 0.22-0.75 | 100 | 0.49 | 0.25-0.89 | 100 | 0.30 | LOD-1.46 | 91 |
| ♀ |  | 0.10 | LOD-0.36 | 56 | 0.15 | LOD-0.58 | 89 | 0.26 | LOD-1.39 | 86 | 0.13 | LOD-0.47 | 68 |
| 0-para |  | 0.04 | LOD-0.18 | - | 0.13 | LOD-0.58 | - | 0.3 | LOD-0.50 | - | 0.18 | LOD-0.47 | - |
| 1-para |  | 0.14 | 0.10-0.14 | - | 0.29 | 0.13-0.29 | - | 0.17 | 0.10-0.46 | - | 0.09 | LOD-0.20 | - |
| 2-para |  | 0.09 | LOD-0.36 | - | 0.16 | LOD-0.35 | - | 0.07 | LOD-0.16 | - | 0.06 | LOD-0.16 | - |
| **SumPFOS** | **0.10** | **17.0** | **9.15-47.4** | **100** | **27.1** | **6.52-63.9** | **100** | **33.1** | **8.51-65.7** | **100** | **13.0** | **3.89-49.1** | **100** |
| ♂ |  | 21.7 | 10.1-47.4 | - | 41.1 | 24.8-63.9 | - | 41.6 | 23.9-53.9 | - | 19.5 | 14.5-49.1 | - |
| ♀ |  | 15.3 | 9.15-34.9 | - | 22.7 | 6.52-53.2 | - | 28.1 | 8.51-65.7 | - | 10.5 | 3.89-24.4 | - |
| 0-para |  | 14.9 | 9.76-34.9 | - | 23.0 | 6.52-53.2 | - | 30.3 | 16.6-34.9 | - | 11.4 | 9.43-24.4 | - |
| 1-para |  | 16.2 | 11.1-17.6 | - | 32.4 | 21.7-33.6 | - | 26.4 | 8.51-31.8 | - | 8.25 | 6.42-12.8 | - |
| 2-para |  | 15.3 | 9.15-21.5 | - | 16.5 | 11.8-29.6 | - | 13.4 | 9.64-65.7 | - | 7.57 | 3.89-8.73 | - |
| **PFOSlin** | **0.10** | **10.2** | **5.31-26.0** | **100** | **14.9** | **4.20-37.1** | **100** | **18.7** | **4.99-36.3** | **100** | **7.29** | **2.53-30.5** | **100** |
| ♂ |  | 13.3 | 5.31-26.0 | - | 22.2 | 13.8-37.1 | - | 23.2 | 12.5-36.3 | - | 9.28 | 7.75-30.5 | - |
| ♀ |  | 8.82 | 5.37-18.0 | - | 13.2 | 4.20-30.2 | - | 16.6 | 4.99-35.8 | - | 6.06 | 2.53-14.3 | - |
| 0-para |  | 8.41 | 5.37-18.0 | - | 13.2 | 4.20-30.2 | - | 17.9 | 10.7-21.9 | - | 6.51 | 5.39-14.3 | - |
| 1-para |  | 10.21 | 7.41-10.7 | - | 17.3 | 13.10-19.7 | - | 14.8 | 4.99-18.7 | - | 4.92 | 3.78-7.15 | - |
| 2-para |  | 8.61 | 5.74-13.3 | - | 9.67 | 7.03-16.4 | - | 12.0 | 6.67-35.8 | - | 4.82 | 2.53-5.99 | - |
| **PFOSBr** | **0.10** | **7.22** | **3.41-21.3** | **100** | **12.2** | **2.32-27.0** | **100** | **14.6** | **2.97-29.9** | **100** | **6.21** | **1.34-19.5** | **100** |
| ♂ |  | 8.13 | 4.77-21.3 | - | 18.5 | 9.87-27.0 | - | 17.5 | 10.5-27.1 | - | 11.4 | 6.77-19.5 | - |
| ♀ |  | 6.34 | 3.41-16.9 | - | 8.96 | 2.32-23.0 | - | 11.5 | 2.97-29.9 | - | 4.31 | 1.34-10.2 | - |
| 0-para |  | 6.53 | 4.39-16.8 | - | 10.1 | 2.32-23.02 | - | 12.1 | 5.90-16.42 | - | 5.25 | 3.62-10.2 | - |
| 1-para |  | 5.97 | 3.66-6.91 | - | 14.4 | 8.60-15.1 | - | 10.5 | 3.52-15.3 | - | 3.74 | 1.79-5.66 | - |
| 2-para |  | 6.71 | 3.41-8.20 | - | 6.83 | 4.77-13.1 | - | 6.33 | 2.97-29.9 | - | 2.05 | 1.34-4.16 | - |
| **PFOA** | **0.05** | **2.45** | **0.94-7.84** | **100** | **3.96** | **1.08-15.1** | **100** | **3.59** | **0.68-7.72** | **100** | **2.63** | **0.81-7.22** | **100** |
| ♂ |  | 3.31 | 1.03-7.84 | - | 4.92 | 3.36-15.1 | - | 4.16 | 2.69-7.72 | - | 3.28 | 1.77-4.85 | - |
| ♀ |  | 1.97 | 0.94-3.98 | - | 3.64 | 1.08-6.97 | - | 2.89 | 0.68-5.31 | - | 2.30 | 0.81-7.22 | - |
| 0-para |  | 1.97 | 1.70-3.98 | - | 3.92 | 1.08-5.81 | - | 3.71 | 1.39-5.31 | - | 3.12 | 1.82-7.22 | - |
| 1-para |  | 2.12 | 1.35-2.65 | - | 3.79 | 2.89-6.97 | - | 2.17 | 0.68-3.10 | - | 1.25 | 0.81-2.04 | - |
| 2-para |  | 1.49 | 0.94-3.05 | - | 2.28 | 1.70-4.55 | - | 1.86 | 1.34-3.26 | - | 1.64 | 0.84-2.30 | - |
| **PFNA** | **0.10** | **0.26** | **LOD-0.64** | **93** | **0.33** | **LOD-0.63** | **93** | **0.40** | **LOD-0.76** | **97** | **0.59** | **0.23-1.47** | **100** |
| ♂ |  | 0.33 | 0.22-0.64 | 100 | 0.46 | 0.23-0.63 | 100 | 0.54 | 0.36-0.76 | 100 | 0.53 | 0.41-1.09 | - |
| ♀ |  | 0.21 | LOD-0.39 | 88 | 0.30 | LOD-0.40 | 89 | 0.29 | LOD-0.75 | 93 | 0.59 | 0.23-1.47 | - |
| 0-para |  | 0.22 | 0.07-0.39 | - | 0.36 | LOD-0.40 | - | 0.28 | LOD-0.57 | - | 0.68 | 0.54-1.47 | - |
| 1-para |  | 0.24 | 0.14-0.32 | - | 0.28 | 0.24-0.35 | - | 0.24 | LOD-0.30 | - | 0.39 | 0.33-0.52 | - |
| 2-para |  | 0.20 | LOD-0.39 | - | 0.25 | LOD-0.37 | - | 0.45 | 0.35-0.75 | - | 0.40 | 0.23-0.60 | - |
| **PFDA** | **0.02** | **0.08** | **LOD-0.14** | **93** | **0.14** | **LOD-0.36** | **93** | **0.24** | **0.06-0.49** | **100** | **0.21** | **0.06-0.59** | **100** |
| ♂ |  | 0.08 | 0.04-0.14 | 100 | 0.19 | 0.11-0.36 | 100 | 0.26 | 0.12-0.49 | - | 0.25 | 0.07-0.51 | - |
| ♀ |  | 0.05 | LOD-0.13 | 88 | 0.15 | LOD-0.26 | 89 | 0.17 | 0.06-0.43 | - | 0.20 | 0.066-0.59 | - |
| 0-para |  | 0.05 | LOD-0.08 | - | 0.16 | LOD-0.26 | - | 0.17 | 0.06-0.43 | - | 0.24 | 0.15-0.59 | - |
| 1-para |  | 0.04 | 0.04-0.10 | - | 0.15 | 0.11-0.24 | - | 0.14 | 0.06-0.24 | - | 0.15 | 0.09-0.21 | - |
| 2-para |  | 0.08 | LOD-0.13 | - | 0.10 | LOD-0.18 | - | 0.27 | 0.09-0.29 | - | 0.13 | 0.06-0.14 | - |
| **PFUnDA** | **0.06** | **0.29** | **LOD-1.02** | **93** | **0.20** | **0.06-0.63** | **100** | **0.28** | **LOD-0.78** | **93** | **0.23** | **LOD-1.12** | **90** |
| ♂ |  | 0.33 | 0.16-1.02 | 100 | 0.22 | 0.10-0.63 | - | 0.31 | LOD-0.78 | 87 | 0.22 | 0.01-0.68 | 100 |
| ♀ |  | 0.26 | LOD-0.11 | 88 | 0.18 | 0.06-0.40 | - | 0.16 | 0.10-0.70 | 100 | 0.23 | LOD-1.12 | 84 |
| 0-para |  | 0.22 | 0.11-0.31 | - | 0.21 | 0.07-0.30 | - | 0.30 | 0.11-0.7 | - | 0.26 | 0.13-1.12 | - |
| 1-para |  | 0.23 | 0.20-0.23 | - | 0.21 | 0.16-0.29 | - | 0.13 | 0.10-0.47 | - | 0.08 | 0.04-0.26 | - |
| 2-para |  | 0.33 | 0.24-0.81 | - | 0.10 | LOD-0.40 | - | 0.18 | 0.17-0.26 | - | 0.13 | LOD-0.24 | - |
|  |  |  |  |  |  |  |  |  |  |  |  |  |  |

^a^n=29 total, N=13 males, N=16 women, N=4 0-para, N=3 1-para and N=9 2-para

^b^n=29 total N= 11 males, N=18 women, N=9 0-para, N=3 1-para N=5 2-para and N=1 3-para

^c^n=29 total, N=15 males, N=14 women, N=6 0-para, N=4 1-para N=3 2-para, and N=1 4-para

^d^n=30 total, N=10 males, N=20 women, N=12 0-para, N=4 1-para and N=4 2-para

**Table S3. Spearman`s rank correlations between PFASs in the years 1986, 1994, 2001 and 2007**

|  | **FOSA** | | | | **PFHxS** | | | | **PFHpS** | | | | | **PFOS** | | | | | **PFOA** | | | | | **PFNA** | | | | | **PFDA** | | | | | **PFUnDA** | | | | |
| --- | --- | --- | --- | --- | --- | --- | --- | --- | --- | --- | --- | --- | --- | --- | --- | --- | --- | --- | --- | --- | --- | --- | --- | --- | --- | --- | --- | --- | --- | --- | --- | --- | --- | --- | --- | --- | --- | --- |
|  | 1986 | 1994 | 2001 | 2007 | 1986 | 1994 | 2001 | 2007 | 1986 | 1994 | 2001 | 2007 | 1986 | | 1994 | 2001 | 2007 | 1986 | | 1994 | 2001 | 2007 | 1986 | | 1994 | 2001 | 2007 | 1986 | | 1994 | 2001 | 2007 | 1986 | | 1994 | 2001 | 2007 |  |
| **FOSA** | - | - | - | - | 0.28 | 0.05 | -0.02 | - | 0.3 | 0.18 | -0.03 | - | 0.57 | | 0.29 | 0.14 | - | 0.48 | | 0.23 | 0.19 | - | 0.14 | | 0.4 | -0.04 | - | 0.05 | | 0.27 | 0.06 | - | 0.35 | | 0.14 | 0.10 | - |  |
| **PFHxS** | 0.28 | 0.05 | -0.02 | - | - | - | - | - | 0.67 | 0.73 | 0.53 | 0.59 | 0.78 | | 0.72 | 0.7 | 0.76 | 0.78 | | 0.79 | 0.5 | 0.64 | 0.77 | | 0.59 | 0.47 | 0.35 | 0.48 | | 0.54 | 0.27 | 0.26 | 0.5 | | 0.47 | 0.46 | 0.25 |  |
| **PFHpS** | 0.30 | 0.18 | -0.03 | - | 0.67 | 0.73 | 0.53 | 0.59 | - | - | - | - | 0.69 | | 0.79 | 0.82 | 0.68 | 0.56 | | 0.72 | 0.59 | 0.45 | 0.66 | | 0.5 | 0.69 | 0.24 | 0.39 | | 0.38 | 0.38 | 0.26 | 0.63 | | 0.28 | 0.44 | 0.09 |  |
| **PFOS** | 0.57 | 0.29 | 0.14 | - | 0.78 | 0.72 | 0.70 | 0.76 | 0.69 | 0.79 | 0.82 | 0.68 | - | | - | - | - | 0.7 | | 0.76 | 0.63 | 0.51 | 0.69 | | 0.74 | 0.76 | 0.3 | 0.38 | | 0.72 | 0.55 | 0.37 | 0.66 | | 0.59 | 0.61 | 0.29 |  |
| **PFOA** | 0.48 | 0.23 | 0.19 | - | 0.78 | 0.79 | 0.50 | 0.64 | 0.56 | 0.72 | 0.59 | 0.45 | 0.7 | | 0.76 | 0.63 | 0.51 | - | | - | - | - | 0.68 | | 0.59 | 0.47 | 0.71 | 0.4 | | 0.41 | 0.28 | 0.41 | 0.41 | | 0.27 | 0.47 | 0.51 |  |
| **PFNA** | 0.14 | 0.40 | -0.04 | - | 0.77 | 0.59 | 0.47 | 0.35 | 0.66 | 0.5 | 0.69 | 0.24 | 0.69 | | 0.74 | 0.76 | 0.3 | 0.68 | | 0.59 | 0.47 | 0.71 | - | | - | - | - | 0.6 | | 0.71 | 0.72 | 0.75 | 0.59 | | 0.64 | 0.6 | 0.76 |  |
| **PFDA** | 0.05 | 0.27 | 0.06 | - | 0.48 | 0.54 | 0.27 | 0.26 | 0.39 | 0.38 | 0.38 | 0.26 | 0.38 | | 0.72 | 0.55 | 0.37 | 0.4 | | 0.41 | 0.28 | 0.41 | 0.6 | | 0.71 | 0.72 | 0.75 | - | | - | - | - | 0.58 | | 0.67 | 0.65 | 0.62 |  |
| **PFUnDA** | 0.35 | 0.14 | 0.10 | - | 0.50 | 0.47 | 0.46 | 0.25 | 0.63 | 0.28 | 0.44 | 0.09 | 0.66 | | 0.59 | 0.61 | 0.29 | 0.41 | | 0.27 | 0.47 | 0.51 | 0.59 | | 0.64 | 0.60 | 0.76 | 0.58 | | 0.67 | 0.65 | 0.62 | - | | - | - | - |  |
